# Various effects of the expression of the xyloglucanase gene from *Penicillium canescens* in transgenic aspen under semi-natural conditions

**DOI:** 10.1186/s12870-020-02469-2

**Published:** 2020-06-03

**Authors:** Elena O. Vidyagina, Natalia M. Subbotina, Vladimir A. Belyi, Vadim G. Lebedev, Konstantin V. Krutovsky, Konstantin A. Shestibratov

**Affiliations:** 1 Branch of the Shemyakin-Ovchinnikov Institute of Bioorganic Chemistry, Russian Academy of Sciences, Prospect Nauki 6, Pushchino, Russian Federation 142290; 2 Institute of Сhemistry, Komi Science Centre, Urals Branch of the Russian Academy of Sciences, Republic of Komi, Pervomaiskaya Str. 48, Syktyvkar, Russian Federation 167000; 3 Department of Forest Genetics and Forest Tree Breeding, George-August University of Göttingen, Büsgenweg 2, 37077 Göttingen, Germany; 4 Center for Integrated Breeding Research, George-August University of Göttingen, Albrecht-Thaer-Weg 3, 37077 Göttingen, Germany; 5 Laboratory of Population Genetics, N. I. Vavilov Institute of General Genetics, Russian Academy of Sciences, Gubkina Str. 3, Moscow, Russian Federation 119991; 6 Laboratory of Forest Genomics, Genomic Research and Education Center, Institute of Fundamental Biology and Biotechnology, Siberian Federal University, Akademgorodok 50a/2, Krasnoyarsk, Russian Federation 660036; 7 Department of Ecosystem Sciences and Management, Texas A&M University, College Station, TX 77843-2138 USA

**Keywords:** Aspen, Gene expression level, Xyloglucanase, *Penicillium canescens*, *Populus tremula*, Transgenic

## Abstract

**Background:**

Recombinant carbohydrases genes are used to produce transgenic woody plants with improved phenotypic traits. However, cultivation of such plants in open field is challenging due to a number of problems. Therefore, additional research is needed to alleviate them.

**Results:**

Results of successful cultivation of the transgenic aspens (*Populus tremula*) carrying the recombinant xyloglucanase gene (*sp-Xeg*) from *Penicillium canescens* in semi-natural conditions are reported in this paper for the first time. Change of carbohydrate composition of wood was observed in transgenic aspens carrying the *sp-Xeg* gene. The transformed transgenic line Xeg-2-1b demonstrated accelerated growth and increased content of cellulose in wood of trees growing in both greenhouse and outside in comparison with the control untransformed line Pt. The accelerated growth was observed also in the transgenic line Xeg-1-1c. Thicker cell-wall and longer xylem fiber were also observed in both these transgenic lines. Undescribed earlier considerable reduction in the wood decomposition rate of the transgenic aspen stems was also revealed for the transformed transgenic lines. The decomposition rate was approximately twice as lower for the transgenic line Xeg-2-3b in comparison with the control untransformed line Pt.

**Conclusion:**

A direct dependence of the phenotypic and biochemical traits on the expression of the recombinant gene *sp-Xeg* was demonstrated. The higher was the level of the *sp-Xeg* gene expression, the more pronounced were changes in the phenotypic and biochemical traits. All lines showed phenotypic changes in the leave traits. Our results showed that the plants carrying the recombinant *sp-Xeg* gene do not demonstrate a decrease in growth parameters in semi-natural conditions. In some transgenic lines, a change in the carbohydrate composition of the wood, an increase in the cell wall thickness, and a decrease in the rate of decomposition of wood were observed.

## Background

Forests play a huge role in the economy as a source of timber and many types of raw materials. The logging is growing every year worldwide [1]. Hardwood trees are of particular interest due to a lower content of resins and lignins, making them a convenient source of raw materials for various industries [2–4]. Aspen (*Populus tremula*) is currently one of the most promising hardwood species, fast-growing, with a wide distribution range. Aspen wood is increasingly used in the pulp, paper, and viscose industries, and for container, oriented strand board, and bioethanol production [5]. However, with a current high rate of logging of hardwood species, especially aspen, a shortage of raw materials is expected. Therefore, it is necessary not only to increase the efficiency and rationality of the use of wood, but also to establish forest plantations with increased productivity based on the new and improved tree forms and varieties. Their creation by traditional breeding is a long and not very efficient process [6], while the genetic engineering approach is considered as one of the promising methods for obtaining trees with desired properties [7].

Increase in plant cell size is possible by enzymatic softening the cell wall followed by its expansion due to increased intracellular pressure [8]. Xyloglucan is involved in formation of a strong cellular framework, a plant cell wall hemicellulose polysaccharide that cross-links nearby cellulose microfibrils [9]. Its scission leads to softening of the cell wall [10]. This has been used to create high-productive woody plants with overexpressed xyloglucan decomposition enzymes. The expected increase in plant growth and a change in the biochemical composition of wood were observed under greenhouse conditions [11, 12]. However, tests in open air are also much needed, and our study presented here helps to fill this gap in our knowledge. For example, Park et al. [12] showed in the climatic chamber experiments that length of stems and discoloration of leaves were increased in white poplar (*Populus alba*) transformed with the xyloglucanase gene *AaXEG2* from *Aspergillus aculeatus* in comparison with the control. There was also an increase in cellulose content and a reduction in hemicellulose in transgenic trees [12]. However, the growth rate of the transgenic trees in the field was lower than that of wild-type control trees [13].

The analysis of the world experience has shown that there are successful results in using of recombinant carbohydrases and xyloglucanases to increase the growth rate and improve the quality of aspen wood (genus *Populus*). However, not all issues in this area are completely solved, especially for trees grown in field conditions. Therefore, the aim of this work is to analyze aspen with altered properties of wood carrying the recombinant xyloglucanase *sp-Xeg* gene from *Penicillium canescens* under semi-natural conditions as a preliminary stage before the field trials.

This article reports successful tests of transgenic aspen trees carrying a recombinant xyloglucanase gene *sp-Xeg* and growing under semi-natural conditions. Effects of xyloglucanase gene incorporation on growth parameters, chemical wood composition and rate of wood decomposition are also presented and discussed.

## Results

### Expression of xyloglucanase

**Fig. 1:**
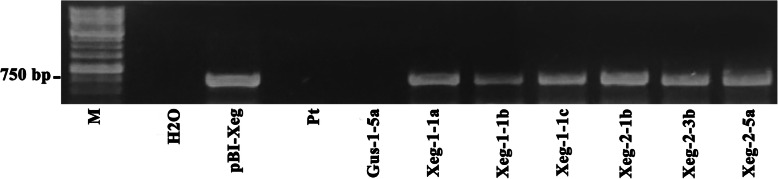
RT-PCR analysis of the *sp-Xeg* gene expression in transgenic aspen plants (expected amplicon size 762 bp). M - standard molecular marker 1 Kb (SibEnzyme Ltd., Russia), Н_2_О - negative reaction control, pBI-Xeg - plasmid DNA (positive control), Pt - non-transgenic control line, Gus-1-5a - transgenic control line. Full-length gel is presented in Supplementary Figure S1

The expression of recombinant gene *sp-Xeg* in the plants growing in semi-natural conditions was confirmed by reverse transcription PCR (RT-PCR) and real-time quantitative PCR (RT-qPCR). The PCR amplification product of the expected size (762 bp) was found in the selected transgenic lines with the inserted xyloglucanase gene, which confirms the presence of transcripts of the recombinant *sp-Xeg* gene (Fig. 1, Additional file 1: Figure S1). The expression data of recombinant and native genes are presented in Table 1.

**Table 1:** Results of the RT-qPCR analysis of the relative *sp-Xeg* gene expression level in the transgenic and control aspen lines

Line	Ct Actin	Ct Ubiquitin	Ct Xeg	∆ Ct	∆∆ Ct	Relative *sp-Xeg* gene expression level
Pt (control)	29.2 ± 0.39	27.5 ± 0.85	30.5 ± 1.78	2.2 ± 2.10	–	–
Xeg-1-1a	30.1 ± 1.45	27.5 ± 0.77	19.1 ± 0.89	−9.7 ± 1.04	−1.4	2.6
Xeg-1-1b	29.9 ± 0.48	25.2 ± 0.33	18.2 ± 0.20	−9.4 ± 0.33	−1.1	2.1
Xeg-1-1c	30.6 ± 1.53	27.5 ± 0.85	18.5 ± 0.95	− 10.6 ± 1.11	−2.2	4.7
Xeg-2-1b	31.1 ± 0.68	27.9 ± 0.48	18.4 ± 0.06	− 11.1 ± 0.40	−2.7	6.7
Xeg-2-3b	28.1 ± 0.43	25.7 ± 0.38	17.3 ± 0.22	− 9.6 ± 0.34	−1.3	2.4
Xeg-2-5a	27.7 ± 0.22	24.4 ± 0.18	17.8 ± 0.30	−8.3 ± 0.23	0	1

The expression level of the recombinant gene was calculated relative to the line Xeg-2-5a with minimal expression of the *sp-Xeg* gene. The maximum level of the *sp-Xeg* gene expression was observed in the Xeg-2-1b line (6.7 folds higher than in Xeg-2-5a) and was significantly higher than in other lines. A very high level of expression of the recombinant gene was also observed in the Xeg-1-1c line (4.7 folds higher than in Xeg-2-5a), while the expression level was much less in the other lines.

**Fig. 2:**
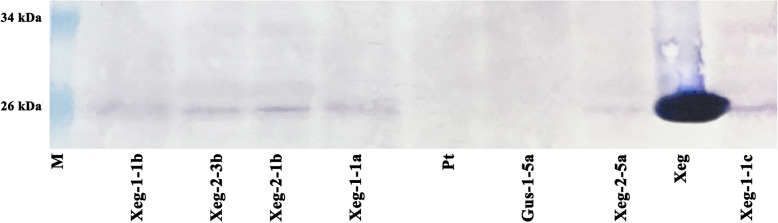
Western blot analysis of protein extracts of transgenic aspens carrying the recombinant gene *sp-Xeg*. M - standard protein molecular marker, Xeg - fungal extract, Pt - non-transgenic control, Gus-1-5a - transgenic negative control, Xeg-1-1a, Xeg-1-1b, Xeg-1-1c, Xeg-2-1b, Xeg-2-3b, and Xeg-2-5a are transgenic lines. Full-length blot is presented in Supplementary Figure S2

Western blotting confirmed the presence of a recombinant XegA protein of the appropriate size (25 kDa) in all six selected transgenic aspen lines carrying the *sp-Xeg* xyloglucanase gene (Fig. 2, Additional file 1: Figure S2). The recombinant protein was detected stably in all replicates of the analysis.

### Growth rate

**Fig. 3:**
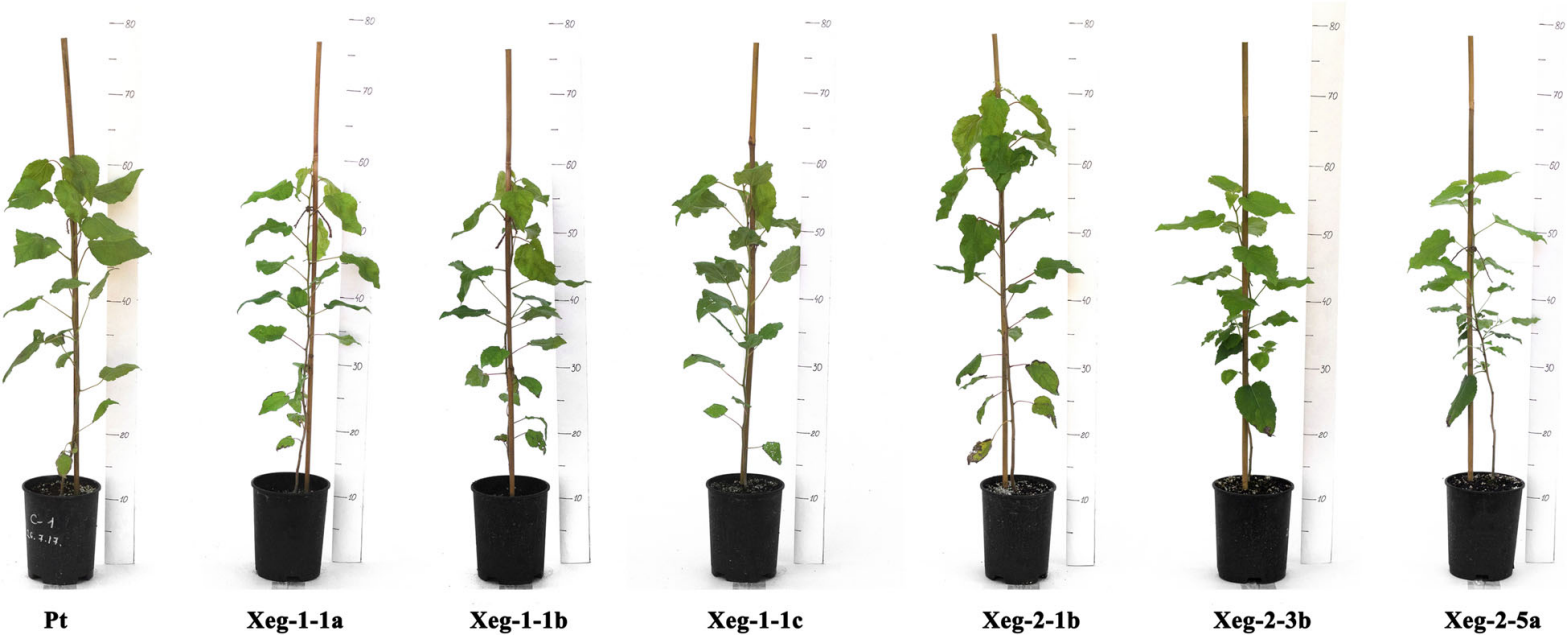
Plant samples of the 18-month-old transgenic and non-transformed control (Pt) lines in semi-natural conditions

**Fig. 4:**
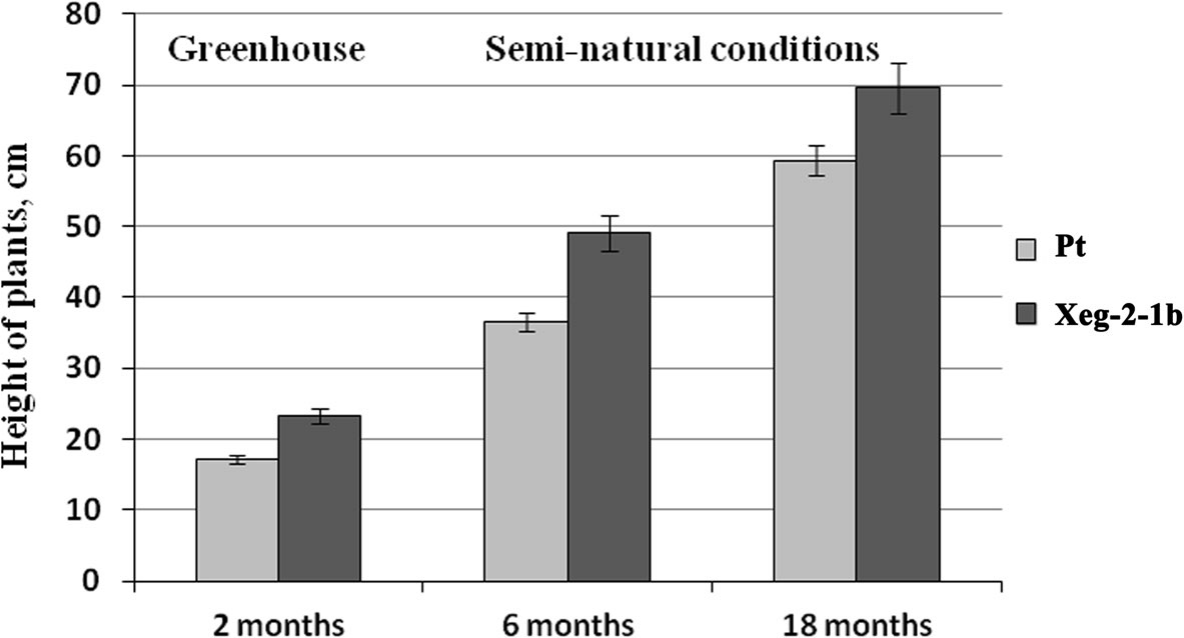
Growth rate of the transgenic (Xeg-2-1b) and non-transgenic control (Pt) aspen lines

Earlier, we noted that in a greenhouse environment, some transgenic lines showed an increase in the growth rate [14]. Transgenic and control plants of aspen were transferred from the greenhouse conditions to an open air and grown under semi-natural conditions up to the age of 18 months. For the analysis, six lines were chosen that demonstrated the presence of a recombinant protein. Four of them had an increased plants height in greenhouse conditions after 2 months in comparison with the Pt control line by 24.6% for the Xeg-1-1a line, 15.7% for Xeg-1-1b, 26.6% for Xeg-2-1b and 15.3% for Xeg-2-3b*.* The other two lines (Xeg-1-1c and Xeg-2-5a) were not different from the control (Additional file 1 in Supplementary information: Table S2). It should also be mentioned that although a tendency in increased tree height was observed for most of the transgenic trees in semi-natural conditions, statistically significant increase in tree height, as well as in stem diameter and volume, was observed only for the Xeg-2-1b line (Table 2, Fig. 3). In the greenhouse, after 2 months of vegetation, this line was taller than non-transgenic Pt control by 26.6%, in the open air after 6 months of vegetation by 25.4%, and after 18 months by 14.6% (Fig. 4).

**Table 2:** Growth rate indicators, number of leaves, and cellulose content (± SD) in the 18-month-old aspen transgenic and control lines in semi-natural conditions

Line	Height, *cm*	Stem diameter, *mm*	Volume, cm^3^	Number of leaves	Cellulose content, *mg/g*
Pt (control)	59.4 ± 1.97	6.6 ± 0.22	25.8 ± 0.95	24.2 ± 0.84	383.2 ± 10.97
Xeg-1-1a	61.1 ± 2.14	6.5 ± 0.15	25.8 ± 0.78	26.2 ± 1.05	381.2 ± 12.33
Xeg-1-1b	53.3 ± 1.91	6.2 ± 0.23	20.5 ± 1.00	25.3 ± 0.88	397.6 ± 11.52
Xeg-1-1c	59.3 ± 2.34	6.9 ± 0.20	28.2 ± 0.93	25.1 ± 0.90	426.4 ± 12.39*
Xeg-2-1b	69.6 ± 2.67*	7.9 ± 0.23*	43.4 ± 1.04*	29.4 ± 0.99*	411.3 ± 11.57*
Xeg-2-3b	51.2 ± 1.85	6.5 ± 0.16	21.63 ± 0.87	25.2 ± 0.82	388.7 ± 11.44
Xeg-2-5a	60.5 ± 2.02	6.1 ± 0.22	22.51 ± 0.98	25.0 ± 1.07	379.6 ± 10.86

*significantly different from Pt at *P* ≤ 0.05 based on ANOVA

Integration of the xyloglucanase gene had little effect on the leaf area of transgenic plants. Only two aspen lines showed a significant decrease in area – Xeg-1-1b by 28% and Xeg-2-3b by 17% compared to the control plants (Table 3).

**Table 3:** Leaf trait parameters (± SD) of the 18-month-old aspen lines

Line	Area, *mm*^*2*^	Circularity	Length, *mm*	Width, *mm*	Length to width
Pt (control)	5776.3 ± 203.9	91.6 ± 1.79	96.3 ± 2.85	81.0 ± 2.51	1.20 ± 0.04
Xeg-1-1a	4921.6 ± 172.2	85.5 ± 1.91*	100.1 ± 3.09	69.1 ± 2.03*	1.47 ± 0.05*
Xeg-1-1b	4176.3 ± 156.7*	86.0 ± 1.85*	89.9 ± 2.77	65.3 ± 2.59*	1.39 ± 0.04*
Xeg-1-1c	5526.3 ± 197.8	87.1 ± 1.09*	102.4 ± 2.35	75.0 ± 2.77	1.38 ± 0.04*
Xeg-2-1b	6020.8 ± 210.7	90.1 ± 2.14	105.1 ± 2.71	77.7 ± 2.56	1.37 ± 0.04*
Xeg-2-3b	4784.6 ± 167.4*	88.2 ± 1.15*	95.5 ± 1.98	68.9 ± 2.09*	1.39 ± 0.05*
Xeg-2-5a	5901.5 ± 212.4	86.6 ± 1.55*	108.8 ± 2.79*	76.2 ± 2.45	1.45 ± 0.05*

*significantly different from Pt at *P* ≤ 0.05 based on ANOVA

**Fig. 5:**
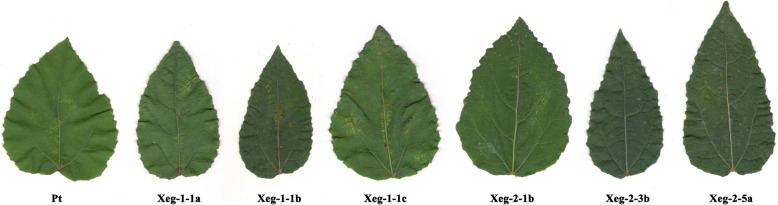
Aspen leaves under semi-natural conditions presenting transgenic and control (Pt) lines

However, almost all transgenic lines changed the leaf shape - they significantly reduced the circularity. This was due to an increase in the length and decrease in the width of the leaf blade of transgenic plants. As a result, all the transgenic lines significantly changed the length-to-width ratio of the leaf by 14–23% compared to the control plants (Fig. 5). For all transgenic plants, there was a tendency to increase the number of leaves, but not statistically significant (Table 2).

### Cellulose content in wood

The content of cellulose was measured in wood of all six studied transgenic lines. A significant increase in cellulose content was observed only in the Xeg-1-1c and Xeg-2-1b lines (Table 2). The content of cellulose in other lines was on average at the control level. The reduction of cellulose content in transgenic lines was not detected. Thus, in terms of cellulose content, the Xeg-1-1c line can be also considered as a prospective line in addition to the Xeg-2-1b line.

### Carbohydrate composition of xylem

Xyloglucanase promotes cleavage of xyloglucan, thereby affecting the carbohydrate composition. A decrease in the content of pentosans (the main component of hemicellulose) was observed in all greenhouse plants [14]. In plants under semi-natural conditions, there was also a decrease in the content of pentosans in all transgenic lines in the youngest parts of the stem in comparison with control (Table 4). Less difference from control was observed in the older (18-month-old) parts of the stem, and four out of six lines demonstrated an increase in the content of pentosans. However, all changes were statistically insignificant.

**Table 4:** The percentage of pentosans (± SD) in the wood of the 6- and 18-month-old aspen lines

Line	6-month-old	18-month-old
Pt	23.1 ± 0.57	18.8 ± 0.56
Xeg-1-1a	17.5 ± 0.53*	17.8 ± 0.48
Xeg-1-1b	20.8 ± 0.46	20.7 ± 0.60
Xeg-1-1c	21.8 ± 0.94	20.3 ± 0.58
Xeg-2-1b	21.5 ± 0.53	17.9 ± 0.50
Xeg-2-3b	21.3 ± 0.76	19.8 ± 0.55
Xeg-2-5a	23.0 ± 0.61	22.2 ± 0.61

* significantly different from Pt at *P* ≤ 0.05 based on ANOVA

**Fig. 6:**
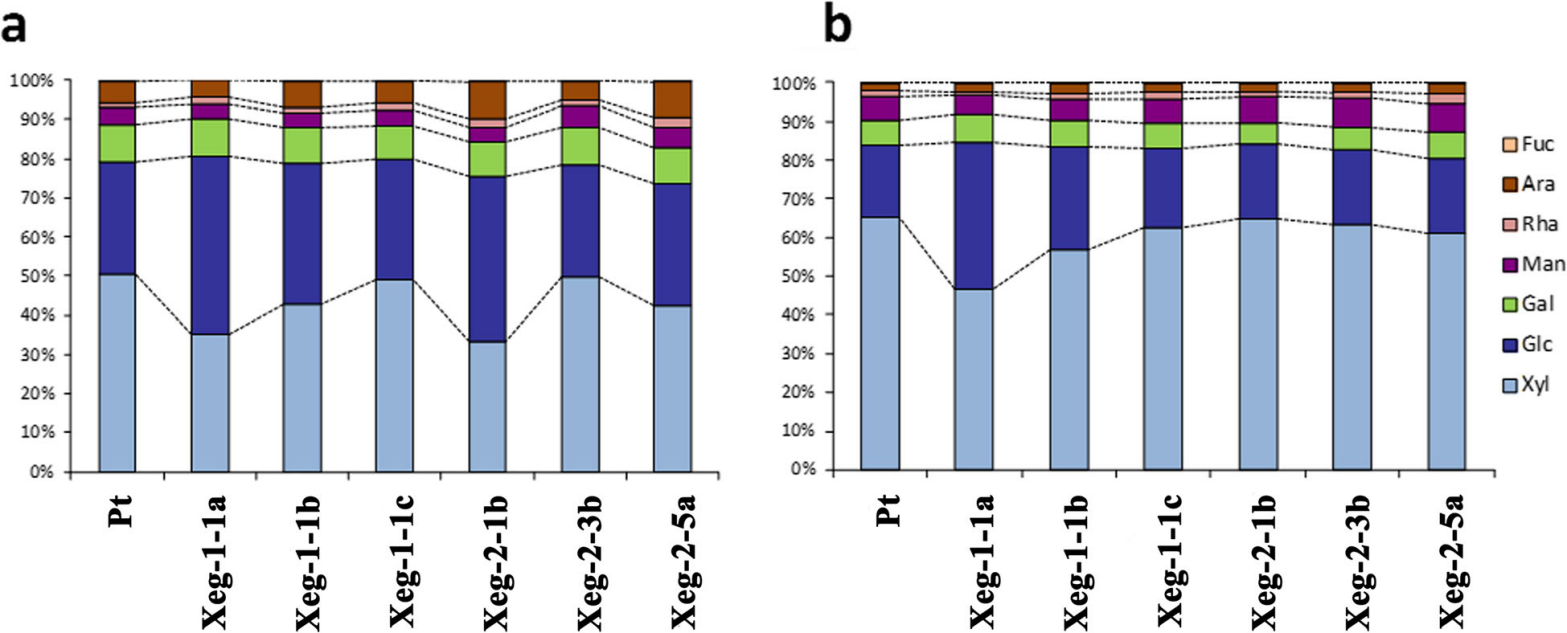
Monomeric sugars composition in wood of the 6- (**a**) and 18- (**b**) month-old transgenic and control (Pt) aspen lines. Fuc – fucose, Ara – arabinos, Rha – rhamnose, Man – mannose, Gal – galactose, Glc – glucose, Xyl – xylose

The ratio of hemicellulose components was also affected in transgenic lines (Fig. 6). In the youngest parts of the plant, a significant decrease in the xylose content was observed in most of the lines, accompanied by an increase in the content of glucose and sometimes arabinose (Fig. 6a). The content of galactose and mannose varied insignificantly. In the most mature wood, the proportions of xylose and glucose did not differ significantly from control, but the content of arabinose and mannose was slightly, but significantly increased (Fig. 6b). The relative content of rhamnose and fucose in aspen wood was relatively low: 1.5–2% of rhamnose, independently of the wood age, and 0.2–0.5% and 0.1–0.2% of fucose in the younger and older wood, respectively.

### Libriform fiber analysis

The diameter and length of the libriform fiber measured in wood samples of six studied lines are presented in Table 5. A slight increase in the fiber diameter was observed in transgenic plants. The fiber length was significantly higher than in the control in two lines Xeg-2-1b and Xeg-2-3b (Table 5).

**Table 5:** Mean diameter and length (± SD) of wood fiber in aspen lines

Line	Mean diameter, *mkm*	Mean length, *mkm*
Pt (control)	19.58 ± 1.84	488.9 ± 12.7
Xeg-1-1a	20.14 ± 1.80	506.7 ± 14.3*
Xeg-1-1b	19.76 ± 1.61	487.6 ± 11.6
Xeg-1-1c	20.16 ± 1.27	461.8 ± 17.4
Xeg-2-1b	20.01 ± 1.93	512.0 ± 10.8*
Xeg-2-3b	19.57 ± 1.54	524.3 ± 13.0*
Xeg-2-5a	21.98 ± 1.93	487.8 ± 15.2

* significantly different from Pt at *P* ≤ 0.05 based on ANOVA

### Microscopy of xylem

**Fig. 7:**
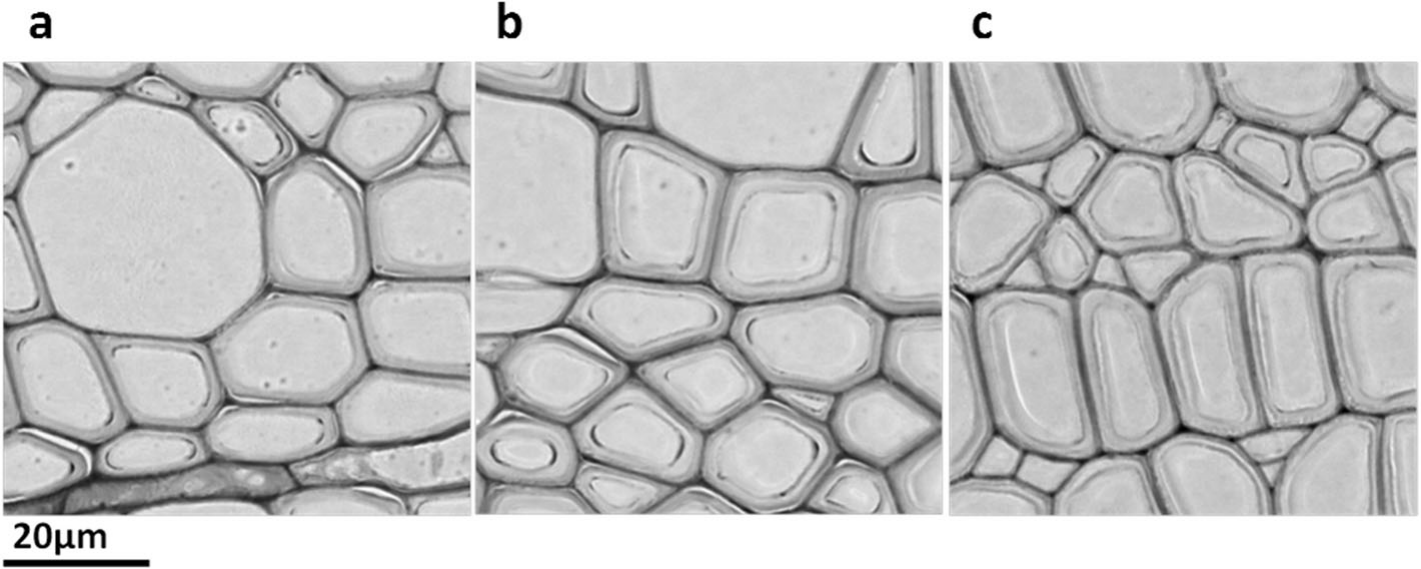
Micrograph of cell slices of plant xylem with different cell wall thickness of 1.16 ± 0.044 μm in the control line Pt (**a**) and 1.47 ± 0.045 and 1.80 ± 0.056 in the transgenic lines Xeg-1-1c (**b**) and Xeg-2-1b (**c**), respectively

Electron microscopy of xylem of 18-month-old aspen showed an increase of the cell wall thickness in prospective lines Xeg-2-1b (1.80 μm on average) and Xeg-1-1c (1.47 μm on average) in the xylem cells of the first year of vegetation in comparison with non-transgenic control (1.16 μm) (Fig. 7; Additional file 1: Figure S3).

### Decomposition rate

**Fig. 8:**
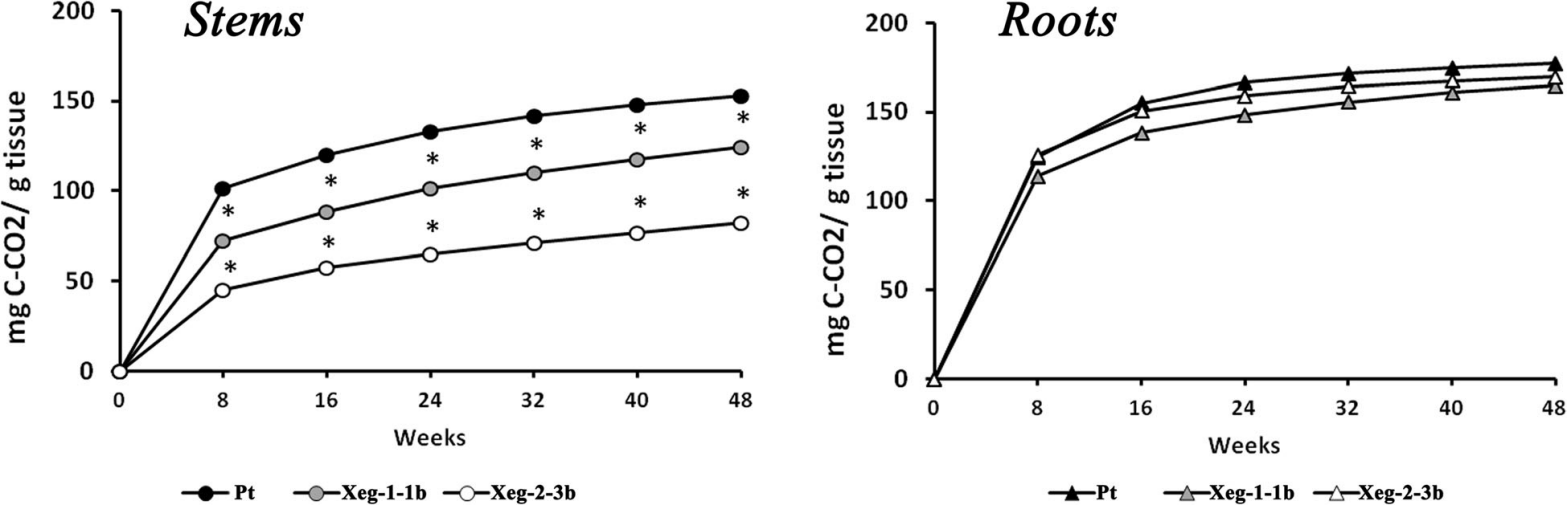
Cumulative CO_2_ emissions during decomposition of stems and roots of transgenic (Xeg-1-1b and Xeg-2-3b) and control (Pt) aspens during the year; *statistically significantly different from Pt at *P* ≤ 0.05 based on ANOVA

Transgenic lines Xeg-1-1b and Xeg-2-3b and control Pt were used in the decomposition experiment. These two transgenic lines were the most morphologically and biochemically similar to Pt. Analysis of the carbon dioxide emission during the decomposition of plant material showed that the stems of transgenic plants had a decomposition rate lower than in the control, especially for Xeg-2-3b, where it was about two times slower than in the control line Pt (Fig. 8). However, during root decomposition no significant difference in the carbon dioxide emission was detected between transgenic and control plants.

A significant decrease of nitrogen was observed in stems of transgenic plants in comparison with the control line, while no change for nitrogen was found in the roots. No change for carbon was found in both roots and stems of the transgenic lines in comparison with the control line (Table 6).

**Table 6:** Percentage of nitrogen and carbon (± SD) in the wood of the aspen lines

Line	Stems		Roots	
	nitrogen	carbon	nitrogen	carbon
Pt	1.23 ± 0.12	45.8 ± 1.5	1.7 ± 0.3	45.5 ± 1.5
Xeg-1-1b	1.04 ± 0.19*	46.1 ± 1.5	1.9 ± 0.4	45.5 ± 1.5
Xeg−2-3b	1.05 ± 0.18*	45.9 ± 1.5	1.8 ± 0.3	44.9 ± 1.4

* significantly different from Pt at *P* ≤ 0.05 based on ANOVA

## Discussion

Studies of the effect of the introduced recombinant construct in transgenic woody plants are usually based on a small sample of the analyzed lines selected from the entire panel of generated transgenic lines. Therefore, it is typical that only six out of 25 transgenic lines were selected for further analysis in open ground conditions in our study. For instance, in a similar study of the recombinant *GS1* gene expression in poplar hybrids (*Populus tremula × P. alba*), only eight transgenic lines were analyzed in field trials [15] selected from 22 initially generated transgenic lines that were grown first under greenhouse conditions [16]. Moreover, a detailed study of the recombinant gene was based only on one line [17]. In another similar study of the *PIP1* aquaporin gene the initial panel of *PIP1*-deficient transgenic *Populus tremula x alba* poplar hybrids included 22 lines, but only six lines were selected for initial tests in the greenhouse [18], and then five of them were selected for the detailed study [19]. In the initial study of the isoprene synthase gene *ISPS* 29 transgenic Grey poplar (*Populus*· × *canescens*) lines were generated [20], but only one line was used in the further semi-natural studies [21]. Therefore, following the common practice in the analysis of transgenic woody plants, six from 25 transgenic greenhouse lines generated in our previous study [14] were selected for this study under semi-natural conditions.

The creation of highly productive woody plants with altered wood properties by increasing the cleavage of xyloglucan was considered in some studies as the most promising direction [12, 22]. However, successful results obtained in climatic chambers and greenhouse were not always confirmed in the field [13]. We tested our transgenic aspen lines with recombinant *sp-Xeg* gene also under semi-natural conditions: they had a closed root system and were grown in an open air during the entire vegetation period. We measured various biochemical and phenotypic parameters in plants with the recombinant *sp-Xeg* gene. One of the most important phenotypic indicators was the faster growth of plants. The increase of height is considered as the main indicator of rapid growth in many similar studies related to selection for the faster growing plants [11, 12, 17, 23]. Measurements of their heights demonstrated that not all of the previously identified promising lines [14] have maintained a higher plant height. Convergence of phenotypic traits between transgenic and control lines was observed. The Xeg-2-1b line was the only line that maintained a high degree of productivity throughout the studies compared to the control. This is probably due to the highest level of expression of the recombinant gene. The expression level increase was maximum in this line (6.7 folds), while the expression level in other lines was much less. The increase in growth rate of the Xeg-2-1b line was relatively high in comparison with data published by other researchers [12]. Transgenic white poplars with xyloglucanase from *A. aculeatus* also tended to increase growth rates, but they were grown in climatic chambers [12]. When these plants were grown under greenhouse conditions, no difference in the growth rate was found [24]. Further field trials of these transgenic poplar plants showed that two transgenic white poplar lines trg300–1 and trg300–2 with overexpression of recombinant xyloglucanase from the fungus *A. aculeatus* showed a decrease in the growth rate and total biomass compared with non-transgenic control, while previously demonstrated an increase in productivity in climatic chambers. It was found that the deterioration in growth rates was due to a change in the transpiration process. In transgenic plants stomata dysfunction was observed [13]. The xyloglucanase *XegA*, which we used, is structurally identical to xyloglucanase from *A. aculeatus* (*AaXEG2*) at 70.46% based on the data from the NCBI database. Therefore, similar effects of the recombinant xyloglucanase *XegA* and xyloglucanase *AaXEG2* on plants were observed. The transgenic plants of the line Xeg-2-1b obtained by us maintained the tendency to increase the growth rate in all experimental conditions. It was observed that incorporation of hormonal control or transcription factor genes can affect the size of plant organs [25]. A similar effect was observed in aspens with xyloglucanase gene in our study. We observed the change in a leaf shape in all transgenic lines - the ratio of length to width increased, mainly due to a decrease in the leaf width (Table 3). The final size of plant organs, such as leaves, is controlled by two processes - cell division and cell expansion [26]. The size change can occur not in both, but only in the one dimension. For instance, the overexpression of the cytochrome P450 gene in *Arabidopsis* increased the length of the leaves without any change of their width [27]. Reducing leaf size is possibly associated with a decrease in the number and / or size of cells [28]. A change in leaf trait parameters was detected in all transgenic lines. We found that a significant increase in the length of the wood fiber was observed in transgenic lines without any change in diameter (Table 4). Since xyloglucan is involved in changing the cell shape in growth and differentiation zones [29], the expression of the xyloglucanase gene could also alter the wall extensibility in the leaf cells, which changed the shape of the cells and subsequently the leaf shape. Moreover, the observed in our study tendency of increasing the number of leaves in transgenic plants may have a compensatory effect on the growth characteristics of these plants [14].

The expected effect of the recombinant xyloglucnase gene was a change in the composition of wood monomeric sugars in all transgenic lines. The main structural motif of xyloglucan in genus *Populus* is a repeating block of the XXXG type consisting of three glucose residues containing the xylose substituent (X) and one unsubstituted glucose residue (G) [30]. In the two-year-old wood of the control aspens, the content was quite similar to this indicator, both in absolute values and in the ratio of xylose, 3.6. However, in young wood areas the ratio was sharply reduced to 1.8, because xylose decreased and cellulose increased simultaneously at the same time. The incorporation of the xyloglucanase gene has altered the composition of the polysaccharide matrix, which primarily resulted in an increase in glucose and a decrease in xylose that caused defects in the formation of xylan. It was most significant in young plants, but was also observed in more mature wood, although to a lesser extent (most significantly in the Xeg-1-1a and Xeg-1-1b lines). This suggests that the incorporation of the xyloglucanase gene promoted the formation of hemicellulose, which is characteristic of the earlier stage of plant development. In the more mature wood, the effect of the xyloglucanase gene was minimal - the content of the main components did not differ much from the control, although the share of the minor components - mannose and arabinose - increased slightly. The third component, galactose, practically did not change. However, we noted significant changes in the relative content of other hemicellulose polysaccharides, which may be due to an indirect effect on the biosynthesis of the secondary cell wall. Our data partially agree with the results of Baba et al. [31], according to which 10% decrease in polysaccharide matrix was noted in the poplar with xyloglucanase gene, as well as a slight decrease in xylose due to galactose and arabinose. Unfortunately, in this work the age of plants was not specified. A significant decrease in xylose was observed in all *Populus deltoides* lines with suppression of the glycosyltransferase gene by interfering RNA [32], where it occurred due to galactose and mannose, but the glucose did not change. The reverse effect was in *Populus euramericana* with interfering RNA on endoglucanase gene, where xylose increased substantially, and glucose was reduced, while other hemicellulose sugars - mannose, galactose, arabinose, rhamnose, and fucose remained unchanged [33]. We found that the incorporation of the xyloglucanase gene in our transgenic aspens led to a decrease in the content of hemicellulose in young wood. In addition, the gene xyloglucanase has changed the composition of xyloglucan, making it more characteristic for an early age. We also showed that this effect is weakened with the age of the plant, and it is practically absent in the second year wood. It is possible that compensatory mechanisms for the formation of hemicellulose are amplified with age, and, in addition, it could be promoted by our plants being grown under semi-natural conditions.

It was demonstrated earlier that the cellulose formation can indirectly affect the binding of microfibrils to each other by xyloglucan filaments, and, therefore, the cleavage of which can probably have a stimulating effect on the cellulose biosynthesis [34]. Our plants had an increase in the cellulose content by 7.4 and 11.3% in the lines Xeg-2-1b and Xeg-1-1c, respectively. Our cellulose content data are in consensus with the data on electron microscopy of xylem of transgenic plants, as well as with the data on libriform measurement. The highest expression of the recombinant *sp-Xeg* gene was also detected in these lines. The length of the fibers has increased in some transgenic lines. This is due to the specific interaction of xyloglucanases with the cell walls resulted to the separation of the cellulose microfibrils that led to the cell size increase due to the increase of intracellular pressure [8]. An increase in the cellulose content may be associated with an increase in the thickness of the cell walls, especially if the gelatinous layer increases [35]. We noted that the thickness of the cell wall of xylem in transgenic plants exceeded the control values and averaged 1.63 μm, whereas in control plants it was 1.16 μm. This is comparable to the increase in cellulose content in these plants.

Changes in the wood composition can affect also biogeochemical processes in ecosystems. In our earlier studies, we measured the decomposition rate of aspen wood in lines with the xyloglucanase gene using the method of mass loss [36]. Significant differences in the decomposition rate between transgenic and control plants were found at the early stages in the transgenic roots, but not in stems. However, this method is not accurate enough, since it does not take into account the coefficient of microbial conversion, which includes the increase in the biomass of microorganisms during decomposition [37]. For a more accurate determination of the decomposition rate in plants, it is recommended to measure the intensity of the CO_2_ emission [38]. In our study we used this method in stems and roots, which are rarely used both in decomposition experiments. According to Zhang et al. [39], the overwhelming number of decomposition experiments were conducted with leaves or needles, and branches and roots are only occasionally used. Meanwhile, leaves and roots constitute a significant proportion of annual litter, and the built-in gene has an effect on the composition of the wood. Although our main experiment was conducted for 2 years, we used 6-month-old samples to study decomposition because they better represent the structure of litter in the natural conditions. According to Freschet et al. [40], 41% of the annual forest litter consists of leaves, 11% of the branches with up to 5 *mm* in diameter and 48% of the roots with up to 2 *mm* in diameter. Measurement of the intensity of the CO_2_ emission showed that for wood of stems of transgenic plants, a significant decrease in the rate of decomposition is characteristic. Such a change may be due to a decrease in the nitrogen content of these plants by 15% on average. A number of studies reported that the nitrogen content was directly related to the decomposition rate of plant residues [39, 41, 42]. The higher nitrogen content increased the rate of decomposition, and the lower content reduced it. Perhaps, for the same reason, differences in the emission of carbon dioxide during the decomposition of the roots of our transgenic plants were not detected. It is likely that an increase in the thickness of the cell wall can also affect the rate of wood decomposition [43].

Our analysis of six transgenic lines under semi-natural conditions showed that the effect of the recombinant gene is observed in all transgenic lines. Thus, based on the results of the aspen tests in semi-natural conditions, it can be concluded that the recombinant *sp-Xeg* gene has a complex effect on the plant organism. Recombinant gene influenced not only the growth parameters of transgenic plants, but also the content of cellulose, plant fiber, and decomposition rate of wood. It should be noted that the phenotypic expression was correlated with the expression level of the recombinant xyloglucanase *sp-Xeg* gene. The most prominent of all transgenic lines was the Xeg-2-1b line with the highest level of expression of the recombinant gene. It maintained an increased growth rate and had a higher cellulose content and a thicker cell wall of xylem fibers under all test conditions. The Xeg-1-1c line differing in many traits was second in terms of the level of the *sp-Xeg* gene expression. An increase in cellulose content and in a thickness of cell wall of xylem fibers were also notable in this line. In the experiment on the decomposition rate of wood, both these transgenic lines were statistically different from the control, but the greatest changes were observed in the Xeg-2-3b line, which had also the greater expression of the recombinant gene. In general, all investigated transgenic lines demonstrated phenotypic and biochemical changes, but their manifestation directly depended on the expression level of the *sp-Xeg* recombinant gene.

Modification of plant properties through genetic transformation should always be assessed for its environmental impact. In particular, how transgenic aspen can affect forest ecosystems and soil. The Xeg-2-1b line was defined as the most promising aspen line being the fastest growing and highly productive. A computer simulation of virtual forest plantations consisted of the Xeg-2-1b line and non-transgenic plants was carried out earlier using the EFIMOD simulation model that can predict carbon and nitrogen flows in forest ecosystems with strong feedback mechanism between soil and stand [44]. This model experiment showed that effect of growing transgenic aspens on carbon and nitrogen flows was not different from effect of non-transgenic plants in controls.

## Conclusions

The representative panel of transgenic aspen lines with the constitutive expression of recombinant xyloglucanase gene *sp-Xeg* from *Penicillium canescens* was analyzed. It was proved that the expression of *sp-Xeg* recombinant xyloglucanase leads to a change of thickness of wood fiber and the plant growth. Electron microscopy showed an increase in cell wall thickness in transgenic lines. Libriform analysis also showed an increase in the length and width of the vascular fiber in transgenic plants. An increase in wood fiber parameters is likely to affect growth. For the first time, it was shown that transgenic aspen plants with the gene of recombinant xyloglucanase of fungal origin under test conditions close to the field (semi-natural conditions) do not demonstrate growth reduction. In transgenic plants, *sp-Xeg* recombinant xyloglucanase alters the composition of carbohydrate-containing substances in wood. The change in the content of cellulose and hemicellulose is confirmed by the data obtained in the analysis of the xylem monomeric sugars composition. A direct dependence of the phenotypic and biochemical expression on the level of the recombinant gene expression was found. The higher was the level of the expression of the *sp-Xeg* gene, the more notable was the phenotypic expression. For the first time, an analysis of carbon dioxide emissions during the decomposition of plant material was carried out. It showed that the stems of transgenic plants had a decomposition rate lower than the control ones. This is a preliminary study based on a relatively small panel of the obtained transgenic lines. Although this study is in agreement with many similar studies of transgenic woody plants based also on a small number of transgenic lines selected from a larger panel of generated transgenes (e.g., [15, 18, 23]), we believe that more studies are needed. However, the presented here study has generated important practical data regarding performance of transgenic lines in open ground conditions (semi-natural and field cultivation conditions) and effects of recombinant xyloglucanase *sp-Xeg* affecting not only productivity, but also other parameters. This study supports the trend in the action of recombinant xyloglucanase *sp-Xeg* observed earlier in greenhouse conditions [14]. We plan to continue monitoring field trials with six selected transgenic lines, as well as to analyze the remaining lines under semi-natural cultivation conditions.

## Methods

### Transgenic aspens

Transgenic aspen lines with the introduced recombinant *sp-Xeg* xyloglucanase gene from the fungus *Penicillium canescens* under the transcriptional control of the 35S promoter and nopaline synthase terminator were analyzed. The *sp-Xeg* gene encodes a chimeric xyloglucanase *XegA* with a white poplar cellulase signal peptide [45]. Original in vitro plant material of *Populus tremula* L. (Pt genotype) has been kindly provided by the Institute of Forestry of the National Academy of Sciences of Belarus (Dr. V. E. Padutov, Gomel, Republic of Belarus) and used as a starting material for genetic transformation. This clone is a diploid form of aspen and characterized by rapid growth and resistance to sound rot. Transgenic plants based on this clone were generated at the Branch of the Shemyakin-Ovchinnikov Institute of Bioorganic Chemistry, Pushchino, Russian Federation [45], and all plant material was propagated there. The in vitro*-*derived trees were adapted to in vivo conditions in the climatic chambers for a month and were grown in greenhouses for a further month. In total, 25 transgenic lines, two control lines - non-transgenic wild-type line (Pt) and a transgenic line with the inserted gene *β*-glucuronidase (Gus-1-5a) were studied, respectively.

Each line (genotype) was represented by 50 plants (ramets). The plants were grown in individual plastic containers with a volume of 1 l (with peat to perlite ratio of 3:1) and after 2 months of growth in the greenhouse moved to semi-natural conditions in an open air with additional watering and feeding. Semi-natural conditions are the cultivation of potted plants outside of the greenhouse [46]. Such growing conditions are close to the field and allow us to estimate the resistance of plants to various biotic and abiotic factors. The tests in open air conditions are long and time-consuming, therefore, a small sample of lines from the entire panel of the obtained transformants is usually used to study the effect of the introduced recombinant construct during the transgenesis of woody plants [47–49]. Following this common practice used in studies of transgenic woody plants we selected six transgenic lines from 25 previously transformed greenhouse plants for our analysis under semi-natural conditions [14]. The selected lines were renamed, and the old and new names are presented in Table S1 (Additional file 1 in Supplementary information). The sampled lines included Xeg-1-1a, Xeg-1-1b, Xeg-1-1c, Xeg-2-1b, Xeg-2-3b, and Xeg-2-5a lines. The selection was based on growth rate data (height) obtained for 2-month-old greenhouse plants and presented in Table S2 (Additional file 1 in Supplementary information). The defective Xeg-2-1c line was excluded, and lines with growth indicators either a significantly higher (Xeg-1-1a, Xeg-2-1b) or similar (Xeg-1-1b, Xeg-1-1c, Xeg-2-3b, Xeg-2-5a) to the control line (Pt) were included.

After 4 months of vegetation under semi-natural conditions, six transgenic lines and a non-transgenic control line (Pt) were selected for the further analysis. Selected lines were transplanted into plastic containers with a volume of 2 l. Nontransgenic control aspens (natural variant) were randomized with aspens of transgenic lines and grown together alongside each other under the same conditions. Under these conditions the plants were grown up to the age of one and a half years with wintering in natural conditions.

After 2 months of growth in the greenhouse and before transfer to semi-natural conditions, the height and number of leaves were measured. After another 4 months of vegetation, growth was measured, samples were taken for molecular analyses and carbohydrate composition measurement, and some of the plants were used to analyze the decomposition rate of the stem wood. The second part wintered and continued to grow for another year, and then, at the age of 18 months, the growth, content of cellulose and pentosans were measured, and samples were taken for carbohydrate composition, microscopy and libriform, and the annual results of the decomposition experiment were evaluated.

The presence of the recombinant gene and protein expression were confirmed in all six transgenic lines. In addition to growth parameters, analyses of specific content of cellulose, pentosans, carbohydrate composition of xylem, measurement of libriform were also performed on all these six transgenic lines and the control line. Electron microscopy was carried out on plants of the Xeg-3 and Xeg-6 lines, which have the greatest differences in the cellulose content compared to the Pt control line. The decomposition rate was measured in the plants of the Xeg-1-1b, Xeg-2-3b and Pt lines. To carry out the decomposition analysis, we selected these two lines that fell into the same group with the control plants according to Duncan’s ANOVA-1 rank tests based on measuring biometric indicators obtained for greenhouse plants, and they differed only by the content of pentosans [14].

Our experimental research complied with institutional, national, or international guidelines. We did not use any endangered species and complied with the Convention on the Trade in Endangered Species of Wild Fauna and Flora.

### Reverse transcription PCR (RT-PCR) analysis

Total plant RNA was extracted from the leaves of all 6-month-old transgenic and control lines growing in the semi-natural conditions by addition of the TRIzol® reagent (Invitrogen, USA) following the manufacturer’s protocol (http://www.invitrogen.com). The purification of RNA, cDNA synthesis, and RT-PCR parameters were described in detail in Vidyagina et al. [14]. However, unlike this previous study 500 ng of total RNA were used for the cDNA synthesis in the study presented here. RNA concentration was measured using a microvolume spectrophotometer Nanodrop 2000 (Thermo Scientific, USA).

### Real-time quantitative PCR (RT-qPCR) analysis

The RT-qPCR method and the analysis of the obtained data were described in detail in Kovalitskaya et al. [50]. Actin and ubiquitin were used as reference genes in the study presented here. Primers used in the RT-qPCR experiments are listed in Table 7.

**Table 7:** Primers used in the RT-PCR

Name	Nucleotide sequence, 5′-3’
XEG-1	ACGGTGTTACCTGGAAGCTG
XEG-2	GACCCTGGTTTTTGATGAGG
Act-1	AAAGTGAAGATATTCAGCCTCTTGT
Act-2	GCGACCCA AATGCTAGG
UBQ-1	GGCAAGACCATAACTCTC
UBQ-2	ATCTTCTAATTGTTTACCAG

### Qualitative Western blot analysis

Total protein extracts were obtained from the leaves of 6-month-old transgenic and control plants ex vitro by the addition of an extraction buffer [0.175 M Tris / HCl (pH 8.8), 5% SDS (w/v), 15% glycerol (v/v), 0.3 M mercaptoethanol] [51]. Protein concentration was determined using a Pierce^TM^ BSA protein assay kit (Thermo Scientific, USA): 120 μg of total protein was added to each well, and 2 μg of xyloglucanase was added to the control well. Electrophoretic separation of proteins was performed according to Laemmli [52] in a 12% polyacrylamide gel. Proteins with molecular weights of 20, 26, 34, 50, 90, and 120 kDa were used as molecular weight markers (Thermo Scientific, Lithuania), and 5 μl of a mixture containg these markers were added to the gel. As a positive control, a xyloglucanase preparation from *P. canescens* was used after three stages of purification (U / mg tamarind seed xyloglucan = 18) and was kindly provided by the IBPM RAS (Pushchino, Russia). Electrotransfer of the gel-separated polypeptides was carried out on a nitrocellulose membrane (Bio-Rad, Germany) using semi-dry transfer (at 40 mA, 2 h) on a TE70PWR translotter (Amersham, USA). To amplify the signal, Pierce^TM^ Western Blot signal enhancer (Thermo Scientific, USA) was used according to the manufacturer’s protocol. Primary polyclonal rabbit antibodies were obtained at the Institute of Theoretical and Experimental Biophysics of the Russian Academy of Sciences (Pushchino, Russia). Hybridization with primary rabbit (1:200) antibodies was carried out for 18 h at 8 °C. Hybridization with goat anti-rabbit antibodies conjugated with alkaline phosphatase (Sigma, USA) was carried out for 2 h at room temperature, with dilution according to the protocol. Immunocomplexes were detected using a BCIP/NBT ready-to-use substrate (Serva, Germany).

### Growth indicators

To study each line, 40 plants were used. The length of the stem was measured from the root neck to the apical bud. Stem diameter was measured at the base of the root neck. The volume (*V*, cm^3^) was measured by the formula: *V = SD*^*2*^ × *H*, where SD - stem diameter (cm), *H* – plant height (cm) [11]. The number of leaves was calculated from the apical bud and to the root neck. The parameters of the leaf blade were measured using the LAMINA software [53] in the second year of vegetation. Height was measured at the age of 2, 6 and 18 months and analyzed using the Statistica 7.0 software (https://www.tibco.com/products/tibco-statistica).

### Analysis of the specific cellulose content

Median internodes of 20 plants per each 18-month-old line were used to determine the content of cellulose by the Kurschner-Hanak nitrogen-alcohol method [54]. The content was recalculated taking into account the weight of an absolutely dry sample.

### Analysis of the specific pentosan content

The specific content of pentosans in wood was estimated using the modified Tollens method [55, 56] by converting them to furfural during distillation in the presence of HCl. The content of pentosans was calculated for dry matter according to Vidyagina et al. [14]. For this analysis, two 10 *cm* long cuts of stem per plant, representing the 1st and the 2nd year growing wood, respectively, were taken from 18-month-old plants.

### Analysis of hemicelluloses monosaccharide

Monomeric sugars of hemicelluloses (arabinose, fucose, galactose, glucose, mannose, rhamnose, and xylose) were measured by standard alditol acetate method [57, 58]. For the analysis of the composition of monosaccharides, two 10 *cm* long cuts of stem per plant without bark, representing the 1st (6 months) and the 2nd (18 months) year growing wood, respectively, were taken from 18-month-old plants. Samples of 5 *mg* of wood sawdust were hydrolyzed with 2 M trifluoroacetic acid (TFA) at 100 °C for 5 h. The mixture of neutral monosaccharides was converted to alditol acetates and identified by gas chromatography–mass spectrometry (GC-MS) analysis using the GCMSQP 2010 Plus chromatograph (Shimadzu Corporation, Japan) with the HP-5MS column (60 *m* × 0.32 *mm* × 0.25 μm). Myo-inositol was used as an internal standard. Helium was used as the carrier gas. The temperature of the injector was 150 °C. The column temperature was increased from 60 °C to 250 °C at a rate of 2 °C/min, and, then, held for 10 min [59].

### Microscopy

For the microscopy analysis, samples of 18-month-old plants were taken from the lower part of the stem (wood of the second year of cultivation) in three replicates. A total cut of all tissues of the stem was made. To prepare the cross sections, the samples were embedded in the epoxy-resin mixture containing DER-332, DER-732, DDSA, and DMP-30 [60]. The transverse sections were obtained using the ultra microtome Reichert Om U2 (Reichert Optische Werke AG, Austria) with glass knives, stained with methylene blue, azur-II, and basic fuchsin [61] and photographed with the AxioImager M1 light microscope (CarlZeiss, Germany). The slices were scanned, and the thickness of the cell walls was estimated using the AxioVision 4.8.1 software package (CarlZeiss, Germany).

### Measurement of the libriform fibers

For the measurement of the libriform fibers, samples of the 18-month-old plants were taken from the bottom of the stem without bark (wood of the second year of cultivation) in three replicates. Samples were macerated using acetic acid and sodium chlorite, and the length and diameter of their libriform fibers were measured [62].

### Measurement of the aspen decomposition rate

The decomposition rate was estimated by analyzing the emission of carbon dioxide during plant material decomposition [63]. In the experiment, sifted through a 0.5 mm fine sieve, washed and sterilized sand was used as a substrate. The plant tissue (stems and roots) from the 6-month-old plants was ground in a porcelain mortar, and, then, dried at 65 °C for 3 days, and 100 mg of this dried and ground tissue were placed in a glass tube with 2 g of sand and sealed with rubber stoppers. To ensure the decomposition in the test tubes, an aqueous extract of the forest plant litter was added. Distilled water was also added to the tubes in an amount of 50% of the total moisture capacity of the sand (taking also into account the water needed to restore the initial mass of plant tissue). Then, the tubes were placed in a thermostat at 22 °C for 48 weeks. Samples of air were sampled from the tubes every 8 weeks, and their carbon dioxide gas was analyzed using a gas chromatograph Crystallux 4000 M (Research and Production Company «Meta-chrom», Yoshkar-Ola, Russia). All samples were analyzed also for C and N content by gas chromatography using the Euro EA-CHNSO Elemental Analyser (HEKAtech GmbH, Wegberg, Germany).

## Supplementary information

**Additional file 1: Table S1** Nomenclature of the transgenic aspen lines used in the study. **Table S2** Height (± SD) in the 2-month-old transgenic and control plants in greenhouse conditions and in the 6-month-old transgenic and control plants in semi-natural conditions. **Figure S1.** The original, unprocessed and uncropped version of Fig. 1. RT-PCR analysis of the *sp-Xeg* gene expression in transgenic aspen plants (expected amplicon size 762 bp). M - standard molecular marker 1 kb (SibEnzyme), Н_2_О - negative reaction control, pBI-Xeg - plasmid DNA (positive control), Pt - non-transgenic control line, Gus-1-5a - transgenic control line. **Figure S2** The original, unprocessed and uncropped version of Fig. 2. Western blot analysis of protein extracts of transgenic aspens carrying the recombinant gene *sp-Xeg*. M - standard protein molecular marker, Xeg - fungal extract, Pt - non-transgenic control, Gus-1-5a - transgenic negative control, Xeg-1-1a, Xeg-1-1b, Xeg-1-1c, Xeg-2-1b, Xeg-2-3b, and Xeg-2-5a are transgenic lines. **Figure S3.** Libriform wood fibers of transgenic and control plants.

## Data Availability

The datasets used and/or analyzed during the current study are available from the corresponding author upon request.

## Abbreviations

*AaXEG2*: Xyloglucanase 2 from *Aspergillus aculeatus*
ANOVA: Analysis of variance
bp: Base pair
cDNA: Complementary DNA
*cel1*: Cellulase gene 1
DNA: Deoxyribonucleic acid
DNase: Deoxyribonuclease
GC-MS: Gas chromatography–mass spectrometry
*GS1*: Glutamine synthetase 1
*ISPS*: Isoprene synthase
kDa: Kilodalton
*P*: Probability
PCR: Polymerase chain reaction
*PIP1*: Aquaporin 1
Pt: *Populus tremula*
RNA: Ribonucleic acid
RNase: Ribonuclease
RT-PCR: Reverse transcription polymerase chain reaction
RT-qPCR: Real-time quantitative polymerase chain reaction
SD: Standard deviation
SDS: Sodium dodecyl sulfate
*sp-Xeg*: Recombinant xyloglucanase from *Penicillium canescens*
TFA: Trifluoroacetic acid
Tris: Tris(hydroxymethyl)-aminomethan
*Xeg*: Xyloglucanase
