# Supplementary material for: Various effects of the expression of the xyloglucanase gene from Penicillium canescens in transgenic aspen under semi-natural conditions
Source: BMC Plant Biol. 2020 Jun 3;20:251. doi: 10.1186/s12870-020-02469-2 (PMC7268456; doi:10.1186/s12870-020-02469-2)
Supplement: Supplementary file 1 — Additional file 1: Table S1 Nomenclature of the transgenic aspen lines used in the study. Table S2 Height (± SD) in the 2-month-old transgenic and control plants in greenhouse conditions and in the 6-month-old transgenic and control plants in semi-natural conditions. Figure S1. The original, unprocessed and uncropped version of Fig. 1. RT-PCR analysis of the sp-Xeg gene expression in transgenic aspen plants (expected amplicon size 762 bp). M - standard molecular marker 1 kb (SibEnzyme), Н2О - negative reaction control, pBI-Xeg - plasmid DNA (positive control), Pt - non-transgenic control line, Gus-1-5a - transgenic control line. Figure S2 The original, unprocessed and uncropped version of Fig. 2. Western blot analysis of protein extracts of transgenic aspens carrying the recombinant gene sp-Xeg. M - standard protein molecular marker, Xeg - fungal extract, Pt - non-transgenic control, Gus-1-5a - transgenic negative control, Xeg-1-1a, Xeg-1-1b, Xeg-1-1c, Xeg-2-1b, Xeg-2-3b, and Xeg-2-5a are transgenic lines. Figure S3. Libriform wood fibers of transgenic and control plants. [file 12870_2020_2469_MOESM1_ESM.zip › Supplementary information_revised2.docx]

**Table S1** Nomenclature of the transgenic aspen lines used in the study

| Old | New |
| --- | --- |
| Pt XIV Xeg 1a | Xeg-1-1a |
| Pt XIV Xeg 1b | Xeg-1-1b |
| Pt XIV Xeg 1c | Xeg-1-1c |
| Pt XIV Xeg 4a | Xeg-1-4a |
| Pt XV Xeg 1a | Xeg-2-1a |
| Pt XV Xeg 1b | Xeg-2-1b |
| Pt XV Xeg 1c | Xeg-2-1c |
| Pt XV Xeg 2а | Xeg-2-2a |
| Pt XV Xeg 2b | Xeg-2-2b |
| Pt XV Xeg 2c | Xeg-2-2c |
| Pt XV Xeg 3a | Xeg-2-3a |
| Pt XV Xeg 3b | Xeg-2-3b |
| Pt XV Xeg 3c | Xeg-2-3c |
| Pt XV Xeg 4a | Xeg-2-4a |
| Pt XV Xeg 4b | Xeg-2-4b |
| Pt XV Xeg 4с | Xeg-2-4c |
| Pt XV Xeg 5a | Xeg-2-5a |
| Pt XV Xeg 5b | Xeg-2-5b |
| Pt XV Xeg 5c | Xeg-2-5c |
| Pt XVI Xeg 1a | Xeg-3-1a |
| Pt XVI Xeg 1b | Xeg-3-1b |
| Pt XVI Xeg 1c | Xeg-3-1c |
| Pt XVI Xeg 5c | Xeg-3-5c |
| Pt XVI Xeg 8a | Xeg-3-8a |
| Pt XVI Xeg 8b | Xeg-3-8b |
| Pt I Gus 5a | Gus-1-5a |

**Table S2** Height (± SD) in the 2-month-old transgenic and control plants in greenhouse conditions and in the 6-month-old transgenic and control plants in semi-natural conditions

| Line | Height, *cm* | |
| --- | --- | --- |
|  | 2-month-old | 6-month-old |
| Pt | 17.1 ± 0.8 | 36.6 ± 1.7 |
| Gus-4-5a | 18.9 ± 1.0 | - |
| Xeg-1-1a | 22.8 ± 1.1* | 45.6 ± 1.2* |
| Xeg-1-1b | 20.2 ± 1.0 | 42.3 ± 1.7 |
| Xeg-1-1c | 19.2 ± 0.9 | 37.6 ± 1.5 |
| Xeg-1-4a | 19.1 ± 0.9 | - |
| Xeg-2-1a | 22.8 ± 1.1* | - |
| Xeg-2-1b | 23.3 ± 0.9* | 48.1 ± 1.8* |
| Xeg-2-1c | 15.7 ± 0.8* | - |
| Xeg-2-2a | 17.1 ± 1.1 | - |
| Xeg-2-2b | 18.3 ± 0.9 | - |
| Xeg-2-2c | 18.5 ± 0.8 | - |
| Xeg-2-3a | 18.3 ± 0.9 | - |
| Xeg-2-3b | 18.6 ± 1.0 | 42.1 ± 1.6 |
| Xeg-2-3c | 18.8 ± 1.1 | - |
| Xeg-2-4a | 19.0 ± 0.9 | - |
| Xeg-2-4b | 18.2 ± 1.0 | - |
| Xeg-2-4c | 17.9 ± 0.9 | - |
| Xeg-2-5a | 17.5 ± 0.9 | 38.2 ± 1.8 |
| Xeg-2-5b | 18.9 ± 0.8 | - |
| Xeg-2-5c | 17.5 ± 0.9 | - |
| Xeg-3-1a | 17.2 ± 0.8 | - |
| Xeg-3-1b | 18.2 ± 0.9 | - |
| Xeg-3-1c | 18.2 ± 0.8 | - |
| Xeg-3-5c | 18.8 ± 0.8 | - |
| Xeg-3-8a | 19.9 ± 0.7 | - |
| Xeg-3-8b | 19.8 ± 1.0 | - |

*significantly different from *Pt* at *P* ≤ 0.05 based on ANOVA


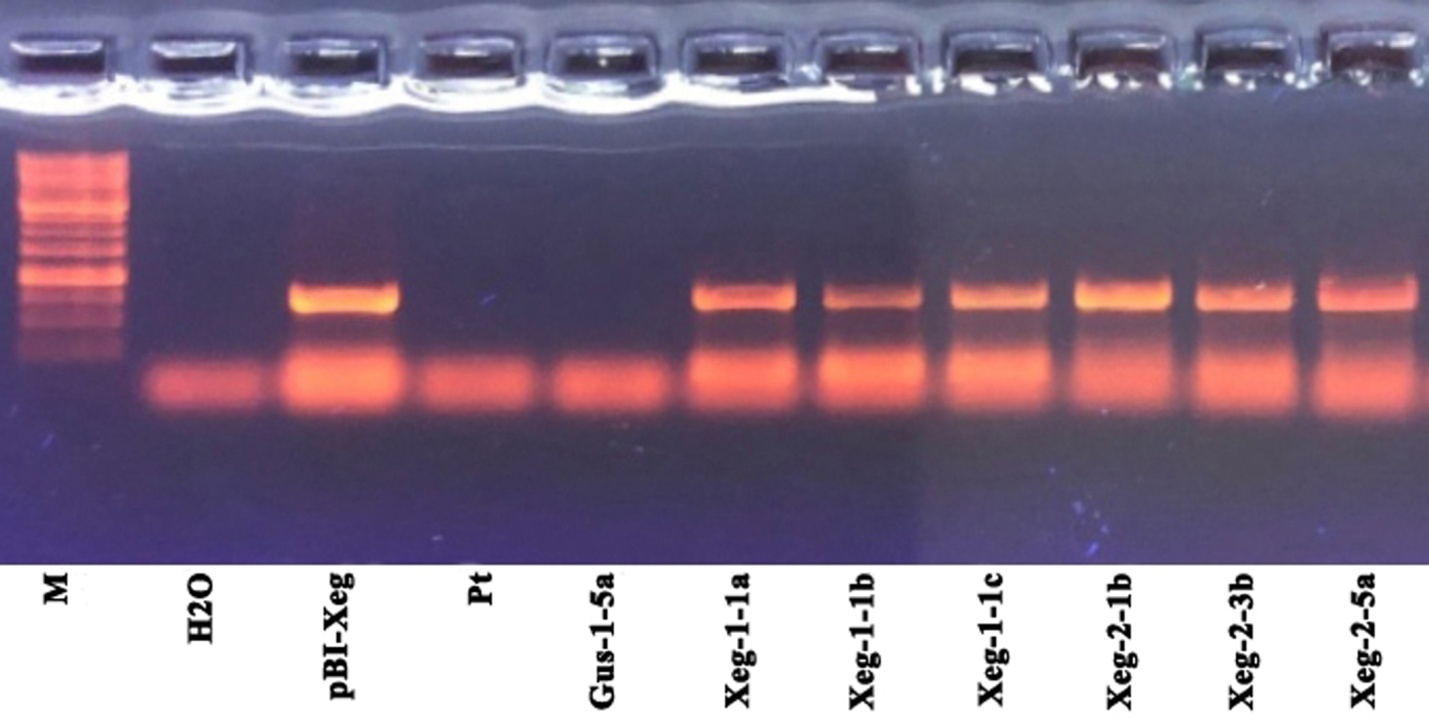


**Figure S1** The original, unprocessed and uncropped version of Figure 1. RT-PCR analysis of the *sp-Xeg* gene expression in transgenic aspen plants (expected amplicon size 762 bp). M - standard molecular marker 1 kb (SibEnzyme), Н_2_О - negative reaction control, pBI-Xeg - plasmid DNA (positive control), Pt - non-transgenic control line, Gus-1-5a - transgenic control line


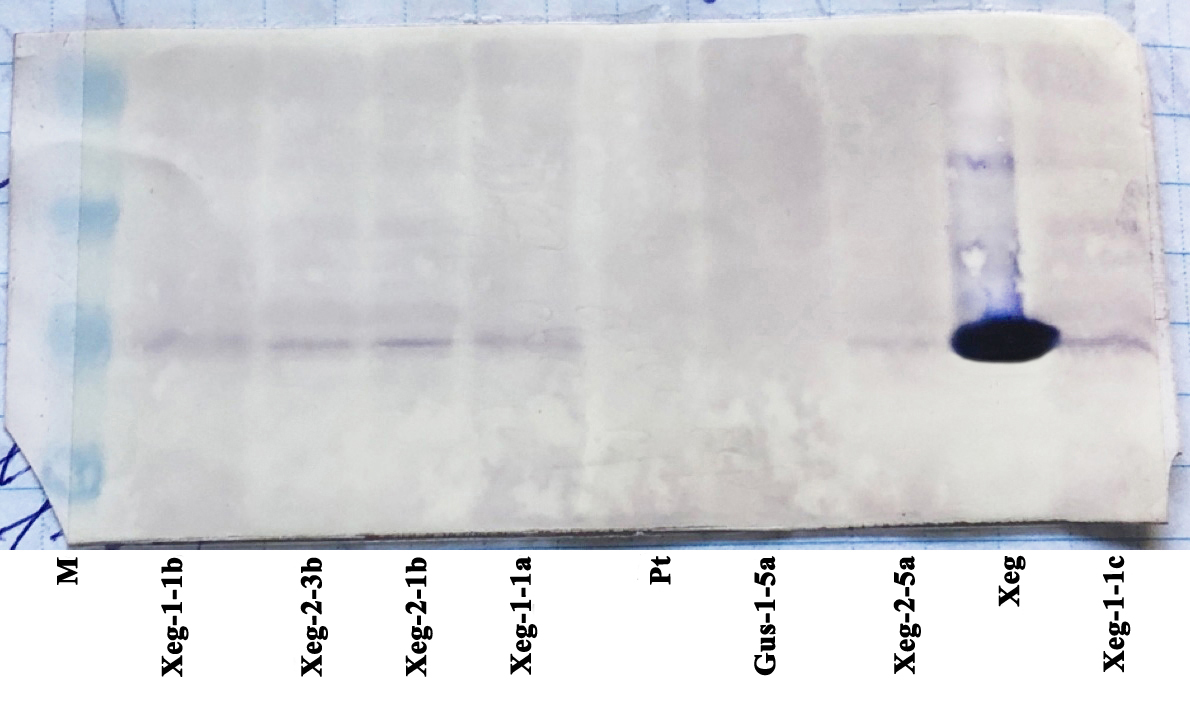


**Figure S2** The original, unprocessed and uncropped version of Figure 2. Western blot analysis of protein extracts of transgenic aspens carrying the recombinant gene *sp-Xeg*. M - standard protein molecular marker, Xeg - fungal extract, Pt - non-transgenic control, Gus-1-5a - transgenic negative control, Xeg-1-1a, Xeg-1-1b, Xeg-1-1c, Xeg-2-1b, Xeg-2-3b, and Xeg-2-5a are transgenic lines

**10.7x zoom**


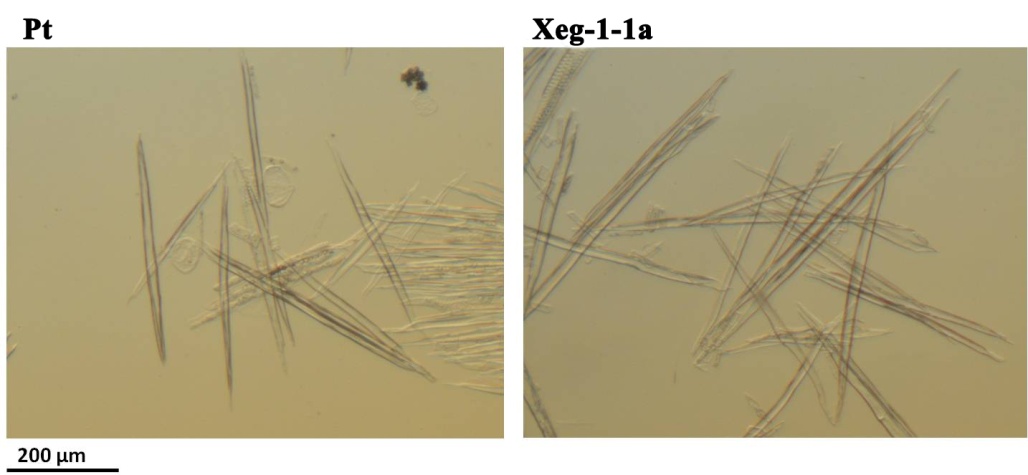

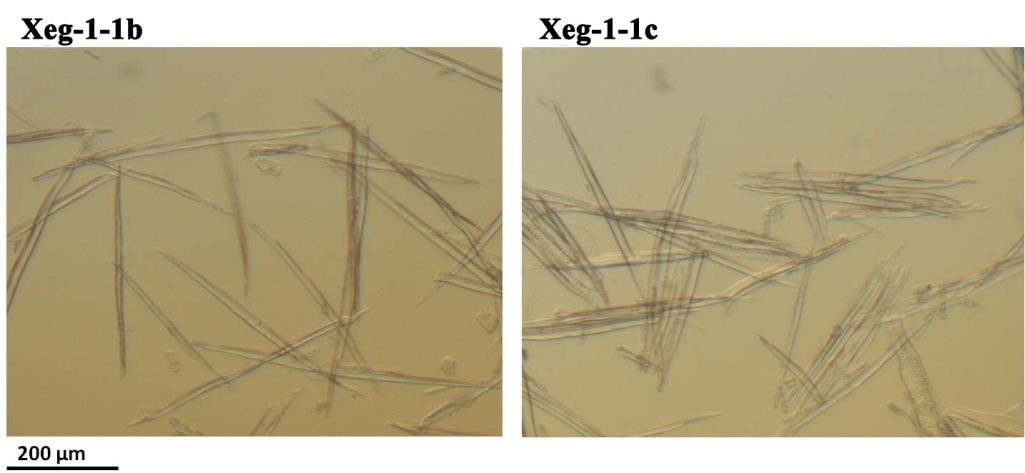


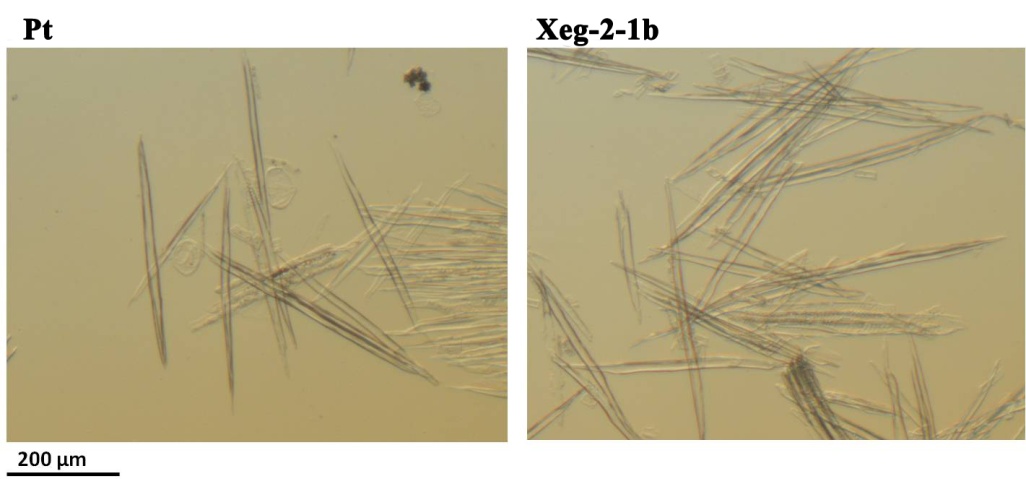

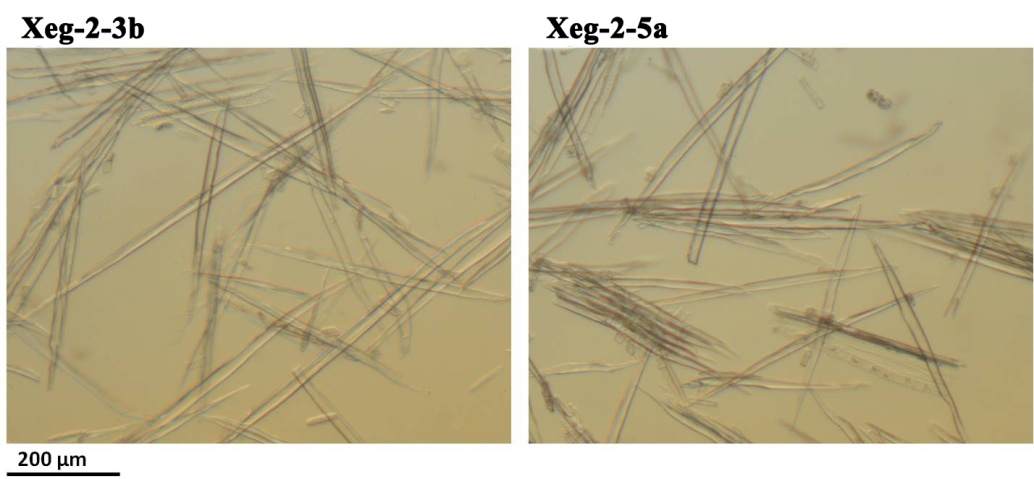


**42.5x zoom**


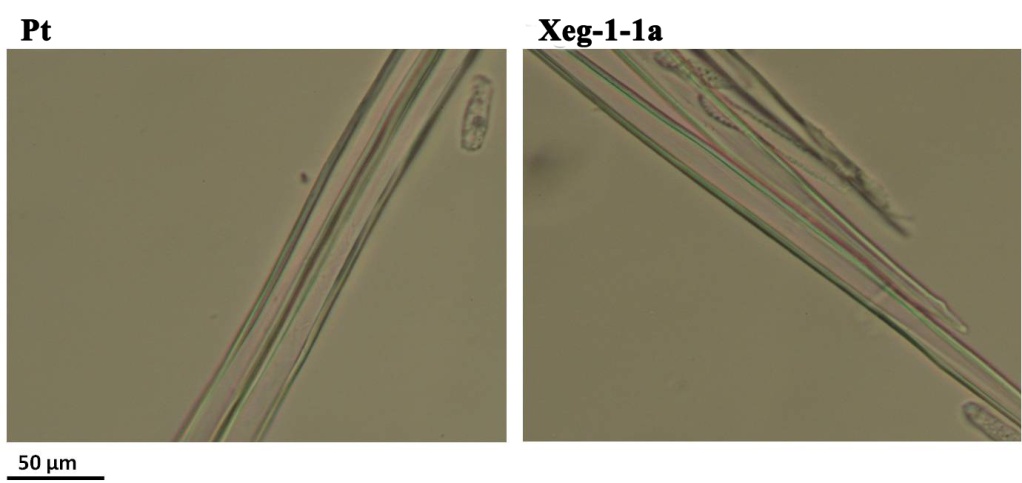

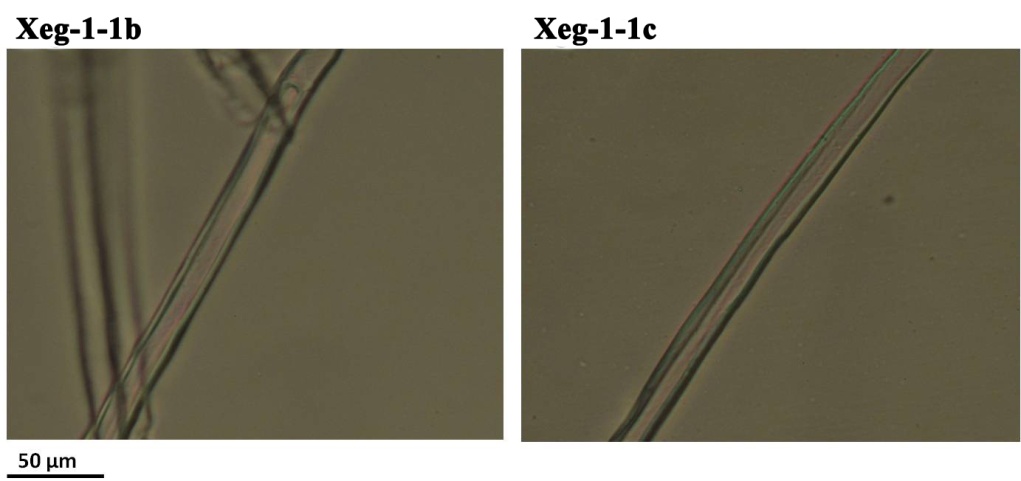

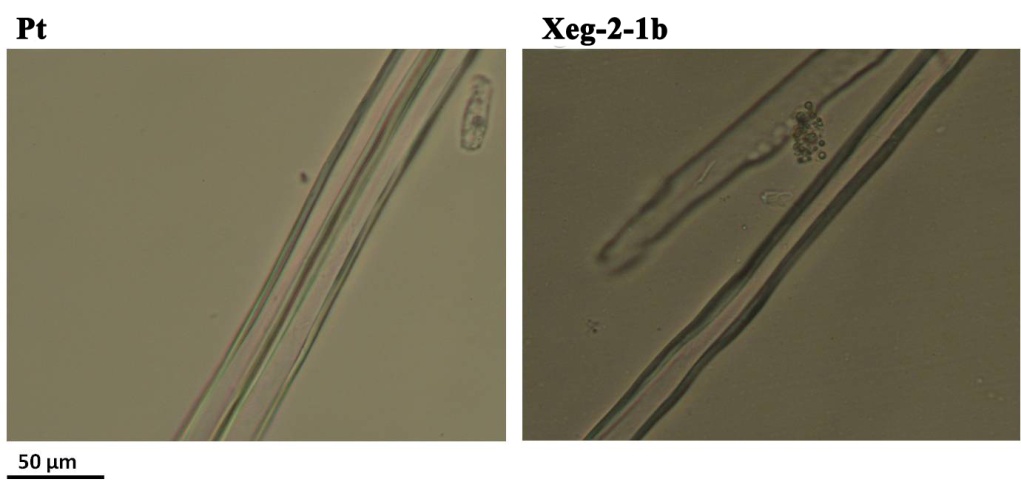

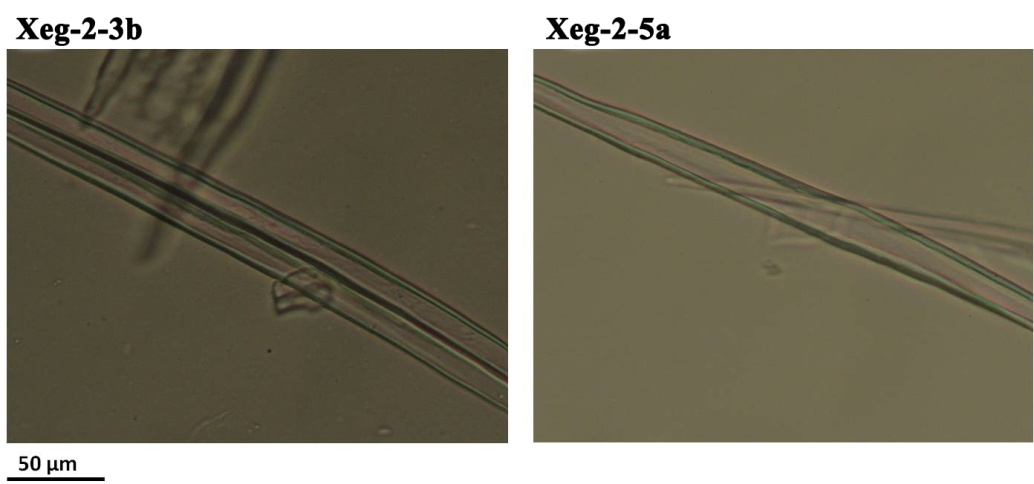


**Figure S3** Libriform wood fibers of transgenic and control plants
