# Supplementary figures and images for: Various effects of the expression of the xyloglucanase gene from Penicillium canescens in transgenic aspen under semi-natural conditions
Source: BMC Plant Biol. 2020 Jun 3;20:251. doi: 10.1186/s12870-020-02469-2 (PMC7268456; doi:10.1186/s12870-020-02469-2)

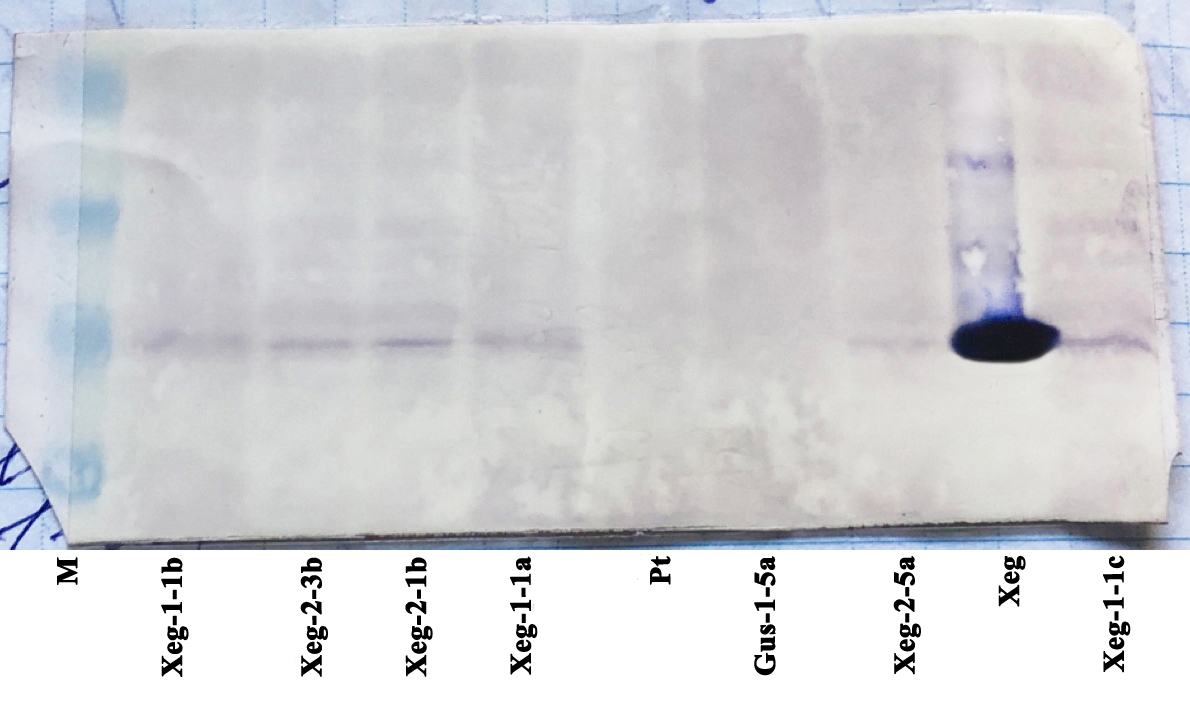

Supplement: Supplementary file 1 — Additional file 1: Table S1 Nomenclature of the transgenic aspen lines used in the study. Table S2 Height (± SD) in the 2-month-old transgenic and control plants in greenhouse conditions and in the 6-month-old transgenic and control plants in semi-natural conditions. Figure S1. The original, unprocessed and uncropped version of Fig. 1. RT-PCR analysis of the sp-Xeg gene expression in transgenic aspen plants (expected amplicon size 762 bp). M - standard molecular marker 1 kb (SibEnzyme), Н2О - negative reaction control, pBI-Xeg - plasmid DNA (positive control), Pt - non-transgenic control line, Gus-1-5a - transgenic control line. Figure S2 The original, unprocessed and uncropped version of Fig. 2. Western blot analysis of protein extracts of transgenic aspens carrying the recombinant gene sp-Xeg. M - standard protein molecular marker, Xeg - fungal extract, Pt - non-transgenic control, Gus-1-5a - transgenic negative control, Xeg-1-1a, Xeg-1-1b, Xeg-1-1c, Xeg-2-1b, Xeg-2-3b, and Xeg-2-5a are transgenic lines. Figure S3. Libriform wood fibers of transgenic and control plants. [file 12870_2020_2469_MOESM1_ESM.zip › Figure2_uncropped.jpg]

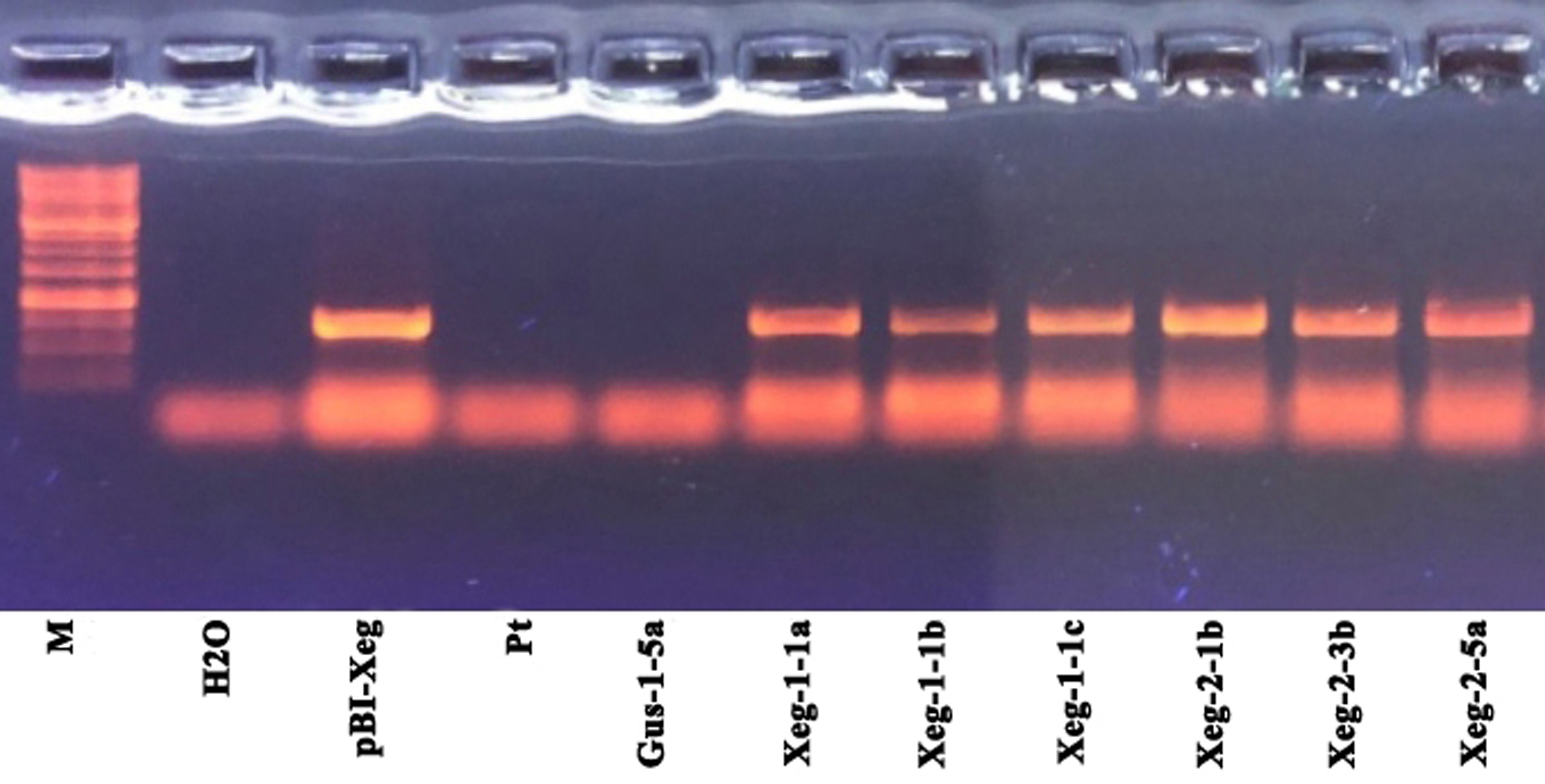

Supplement: Supplementary file 1 — Additional file 1: Table S1 Nomenclature of the transgenic aspen lines used in the study. Table S2 Height (± SD) in the 2-month-old transgenic and control plants in greenhouse conditions and in the 6-month-old transgenic and control plants in semi-natural conditions. Figure S1. The original, unprocessed and uncropped version of Fig. 1. RT-PCR analysis of the sp-Xeg gene expression in transgenic aspen plants (expected amplicon size 762 bp). M - standard molecular marker 1 kb (SibEnzyme), Н2О - negative reaction control, pBI-Xeg - plasmid DNA (positive control), Pt - non-transgenic control line, Gus-1-5a - transgenic control line. Figure S2 The original, unprocessed and uncropped version of Fig. 2. Western blot analysis of protein extracts of transgenic aspens carrying the recombinant gene sp-Xeg. M - standard protein molecular marker, Xeg - fungal extract, Pt - non-transgenic control, Gus-1-5a - transgenic negative control, Xeg-1-1a, Xeg-1-1b, Xeg-1-1c, Xeg-2-1b, Xeg-2-3b, and Xeg-2-5a are transgenic lines. Figure S3. Libriform wood fibers of transgenic and control plants. [file 12870_2020_2469_MOESM1_ESM.zip › Figure1_uncropped.jpg]
